# Development of a multi-locus sequence typing system helps reveal the evolution of *Cardinium hertigii*, a reproductive manipulator symbiont of insects

**DOI:** 10.1186/s12866-019-1638-9

**Published:** 2019-11-27

**Authors:** Corinne M. Stouthamer, Suzanne E. Kelly, Evelyne Mann, Stephan Schmitz-Esser, Martha S. Hunter

**Affiliations:** 1 Department of Entomology, University of Arizona, 410 Forbes Building, Tucson, AZ 85721 USA; 2 Milk Technology and Food Science, Institute for Milk Hygiene, University of Veterinary Medicine, Vienna, Austria; 3 Department of Animal Science, Iowa State University, Ames, Iowa USA

**Keywords:** Endosymbiont, *Wolbachia*, Cytoplasmic incompatibility, Parthenogenesis induction, Feminization, MLST, Phylogenetics

## Abstract

**Background:**

*Cardinium* is an intracellular bacterial symbiont in the phylum Bacteroidetes that is found in many different species of arthropods and some nematodes. This symbiont is known to be able to induce three reproductive manipulation phenotypes, including cytoplasmic incompatibility. Placing individual strains of *Cardinium* within a larger evolutionary context has been challenging because only two, relatively slowly evolving genes, 16S rRNA gene and Gyrase B, have been used to generate phylogenetic trees, and consequently, the relationship of different strains has been elucidated in only its roughest form.

**Results:**

We developed a Multi Locus Sequence Typing (MLST) system that provides researchers with three new genes in addition to Gyrase B for inferring phylogenies and delineating *Cardinium* strains. From our *Cardinium* phylogeny, we confirmed the presence of a new group D, a *Cardinium* clade that resides in the arachnid order harvestmen (Opiliones). Many *Cardinium* clades appear to display a high degree of host affinity, while some show evidence of host shifts to phylogenetically distant hosts, likely associated with ecological opportunity. Like the unrelated reproductive manipulator *Wolbachia*, the *Cardinium* phylogeny also shows no clear phylogenetic signal associated with particular reproductive manipulations.

**Conclusions:**

The *Cardinium* phylogeny shows evidence of diversification within particular host lineages, and also of host shifts among trophic levels within parasitoid-host communities. Like *Wolbachia*, the relatedness of *Cardinium* strains does not necessarily predict their reproductive phenotypes. Lastly, the genetic tools proposed in this study may help future authors to characterize new strains and add to our understanding of *Cardinium* evolution.

## Background

The life histories and evolution of many multicellular organisms are intimately entwined with the microbes they carry [1]. A large number of arthropods carry maternally inherited, intracellular bacterial symbionts that can affect their host’s reproductive outcomes in both detrimental and beneficial ways [2, 3]. These symbionts come from various bacterial phyla, but are categorized based on their associations with their hosts. Primary (or obligate) symbionts complement their hosts’ diet with essential amino acids or other limiting nutrients, are often housed in specialized structures, and are essential to their host’s reproduction (reviewed in Moran et al [2]). Secondary (or facultative) symbionts, though largely unnecessary for successful host reproduction, can provide conditional benefits to their host, have no measurable effect, or manipulate their host’s reproduction in ways that increase the spread of the symbiont [4–6].

Symbiont phylogenies may offer clues to the relationship between the symbionts and their hosts. For instance, primary symbionts, such as *Buchnera* in their aphid hosts, display congruent phylogenies [7], indicating the long evolutionary history and cospeciation of these groups. Secondary symbionts generally have shorter associations with their hosts and may occur at intermediate frequencies within the host population [2]. The evolutionary phylogenies of secondary symbionts generally display many host switches and are non-congruent with their host’s phylogenies (e.g. [8]). Genera of bacteria commonly thought of as secondary symbionts may also include lineages of primary symbionts in their midst, as with *Serratia symbiotica* in aphids [9, 10]. Even the best-known secondary symbiont, *Wolbachia*, a notorious host switcher, contains a clade of symbionts that display congruent evolution and co-cladogensis in their obligatory symbiosis with nematodes [11, 12] as well as a lineage that is required for B-vitamin production in bedbugs [13]. These patterns show that different strains within one group of secondary symbionts can differ dramatically in their relationships with their hosts.

While transitions from secondary to obligate symbiosis may be apparent in phylogenies, as shown by host and symbiont phylogenetic congruence, subtler facets of secondary symbiont life histories may also be elucidated by a well-resolved phylogeny. Horizontal transmission of secondary symbionts between hosts is key to the secondary symbiont lifestyle, yet these transmission events are rarely captured in experiments (see exceptions in Huigens et al. [14] and Caspi-Fluger et al. [15]), and are likely to happen infrequently in nature. Phylogenies are currently the most powerful tools we have to describe these host switches. Well resolved phylogenies may also elucidate co-cladogenesis over a short evolutionary time scale, which can occur when a reproductive manipulator in essence “hijacks” a key reproductive function of their host, creating host-symbiont dependency [6, 16, 17]. In this paper, we explore evolution of the secondary symbiont of arthropods, *Cardinium hertigii* (Bacteroidetes), and address questions concerning horizontal transmission and the evolution of reproductive manipulations with a well-resolved phylogeny.

*Cardinium hertigii*, a member of the phylum Bacteroidetes, infects approximately 7–9% of arthropods [18–20] as well as at least one lineage of the plant parasitic nematode, *Heterodera glycines* [21, 22]. Although it infects many insects, particularly members of Hymenoptera and Hemiptera, much of the diversity of this symbiont genus as described so far appears to lie in arachnids, such as mites, spiders, and harvestmen as hosts [18, 23, 24]. Although the phenotype of *Cardinium* in many hosts is unknown, it has been shown to manipulate host reproduction in insects and mites, and rivals *Wolbachia* in its versatility. Strains of *Cardinium* induce at least three reproductive manipulations: parthenogenesis, feminization, and cytoplasmic incompatibility (CI).

In symbiont-induced parthenogenesis, genetic males turn into genetic females during embryogenesis. Parthenogenesis has been shown or associated with *Cardinium* infection in several parasitoid wasps in the genus *Encarsia* [20, 25] and with the oleander scale, *Aspidiotus nerii* [26]. In feminization, as has been shown in *Brevipalpus* mites, *Cardinium* causes infected genetic males to be converted into functional females [27]. Finally, *Cardinium* is able to induce cytoplasmic incompatibility in several wasps, mites, planthoppers and a thrips [28–35], where infected females produce both male and female offspring, but uninfected females mated with infected males produce few or no offspring (in diploid systems) or few or no daughters (in haplodiploid systems). Of all reproductive manipulators, so far only *Cardinium*, *Wolbachia*, and a recently discovered Alphaproteobacterium [36] have been found to induce CI, although genomic evidence of the *Cardinium* strain *c*Eper1, found in the parasitic wasp *Encarsia suzannae*, suggest that at least *Wolbachia* and *Cardinium* independently evolved this trait [37]. In addition to the reproductive manipulations, *Cardinium* has been shown to affect other host fitness traits as well. In the planthopper *Sogatella furcifera*, *Cardinium* infection is associated with faster nymphal developmental times [34] and in the parasitoid wasp *Encarsia inaron*, *Cardinium* infection is associated with increased longevity of female wasps [38].

Despite the diverse impacts *Cardinium* can have on key aspects of its host’s survival and reproduction, few resources have been devoted towards developing better genetic tools for assessing the evolutionary history of this genus, leaving open some intriguing questions about the symbiont’s evolution and ecological interactions with its hosts. Some of the enduring mysteries involving secondary symbionts, and *Cardinium* in particular, are how these reproductive manipulations evolved. For example, are the genes coding for these manipulations largely horizontally transmitted between strains or do they evolve independently, perhaps repeatedly, within lineages? Additionally, *Cardinium* horizontal transmission rate at a genus-wide level is poorly understood. With weakly resolved phylogenies, it is not clear whether *Cardinium* displays the same low level of host affinity as most other secondary symbionts, or whether the shorter list of host taxa with which it is associated than, for example, the cosmopolitan *Wolbachia,* is indicative of fewer host switches among host lineages. While a total of six *Cardinium* genomes have now been sequenced [39–44] genetic resources that enable broad comparisons among many taxa are still needed. We present four sets of primers from single locus housekeeping genes that each amplify 450-700 bp of DNA in order to more fully resolve the evolutionary relationships of the divergent *Cardinium* strains. By providing primers for the community of *Cardinium* researchers to use to diagnose *Cardinium* and discriminate among as yet uncharacterized strains, the study provides a framework for future studies of this versatile symbiont.

## Results

### MLST primers

Most of the arthropod *Cardinium* in our set of host taxa (Table 1) could be amplified by the MLST primers (Table 2), including members from groups A (the largest arthropod group), C (biting midges in the *Culicoides* group), and D (Opiliones group). All primers amplified products for *Cardinium* residing in Opiliones and *Culicoides* spp. The *EF-G* primers worked on all samples, the *SufB* and *GyrB* primers worked on most samples in group A and all in group C and E. For the *GroEL* primers, two sets of forwards were used (Table 2), depending on which amplified better, but only sequences from the inner forward primer (groel_346F) were used for the phylogenies.

**Table 1:** Collection localities of *Cardinium* strains and their associated reproductive phenotypes

Host organism	*Cardinium* strain	Collection information	Reproductive phenotype
*Aleurothrixus floccosus*	*c*Aflo1	Israel	Unknown
*Encarsia suzannae*	*c*Eper1	Texas, USA	CI (Hunter et al., 2003)
*Encarsia hispida*	*c*Ehis1	San Diego, USA	PI (Zchori-Fein et al., 2004)
*Encarsia tabacivora*	*c*Eper2	Brazil	PI association (Zchori-Fein et al., 2001)
*Encarsia inaron* (IT), high density strain (HIT)	*c*Eina2	Italy	One of two strains co-infecting a host with a CI phenotype (Gebiola et al., 2016). This strain does not cause CI (Stouthamer, et al. unpubl.)
*Encarsia inaron*, (IT), low density strain (LIT)	*c*Eina3	Italy	One of two strains co-infecting a host with a CI phenotype (Gebiola et al., 2016). This strain causes CI (Stouthamer et al. unpubl.)
*Encarsia inaron* (USA)	*c*Eina1	USA	No CI, no PI (White et al., 2009)
*Aspidiotus nerii*	*c*Aner1	University of California, Riverside culture	Associated with parthenogenetic host (Provencher et al., 2005)
*Bemisia tabaci,* Q1 species	*c*BtQ1	Valencia, Spain	No CI, no PI (Fang et al., 2014)
*Ixodes scapularis* cell line	*c*Isca1	Nantucket Island (Massachusetts), USA	Unknown
*Indozuriel dantur*	*c*Idan1	Japan	Unknown
*Sogatella furcifera*	*c*Sfur1	China	Unknown
*Sogatella furcifera*	*c*Sfur2	Japan	CI (Nakamura et al., 2009)
*Eotetranychus suginamensis*	*c*Esug1	Taiwan	CI (Gotoh et al., 2007)
*Oligonychus coffeae*	*c*Ocof1	Japan	Unknown
*Oligonychus gotohi*	*c*Ogot1	Japan	Unknown
*Panonychus mori*	*c*Pmor1	Japan	CI (Gotoh et al., 2003)
*Tetranychus pueraricola*	*c*Tpue1	Japan	No CI, no PI (Gotoh et al., 2003)
*Culicoides arakawae*	*c*Cara1	Kagoshima Pref. or Okinawa Pref., Japan	Unknown
*Culicoides ohmorii*	*c*Cohm1	Kagoshima Pref, Japan	Unknown
*Culicoides peregrinus*	*c*Cper1	Yonaguni Isl., Okinawa Pref. Japan	Unknown
*Culicoides punctatus*	*c*Cpun1	Leahurst Campus, University of Liverpool, UK	Unknown
*Cybaeus eutypus*	*c*Ceut	Vancouver Island, Canada	Unknown
*Cybaeus signifer*	*c*Csig1	Vancouver Island, Canada	Unknown
*Cybaeus chauliodus*	*c*Ccha1	Northern California, USA	Unknown
*Cybaeus somesbar*	*c*Csom1	Northern California, USA	Unknown
*Cybaeus sanbruno*	*c*Csan1	North central California, USA	Unknown
*Cybaeus morosus*	*c*Cmor1	British Columbia, Canada	Unknown
*Cybaeus hesper*	*c*Ches1	North central California, USA	Unknown
*Cybaeus multnoma*	*c*Cmul1	Oregon, USA	Unknown
*Cybaeus penedentatus*	*c*Cpen1	North central California, USA	Unknown
*Culicoides imicola*	*c*Cimi1	Unknown	Unknown
*Metaseiulus occidentalis*	*c*Mocc1	Washington and Oregon, USA	CI (Roush and Hoy, 1981)
*Leiobunum sp 1*	*c*Lsp2	Georgetown Island, Maine, USA	Unknown
*Leiobunum sp 2*	*c*Lsp3	N. Monmouth, Maine, USA	Unknown
*Leiobunum*	*c*Lsp1	Ellison Park, Monroe County, New York, USA	Unknown
*Brevipalpus californicus*	*c*Bcal1	Minas Gerais, Brazil	Feminization (Groot and Breeuwer, 2006)
*Brevipalpus phoenicis*	*c*Bpho1	Minas Gerais, Brazil	Feminization (Groot and Breeuwer, 2006)
*Macrosteles quadrilineatus*	*c*Mque1		Unknown
*Encarsia perniciosi*	*c*Eper3	Tijuana River Valley Park, San Diego, USA	Associated with parthenogenetic host (Stouthamer and Luck, 1991)
*Pezothrips kellyanus*	*c*Pkel1	Australia	CI (Nguyen et al., 2017)

### Phylogenetic trees

**Fig. 1:**
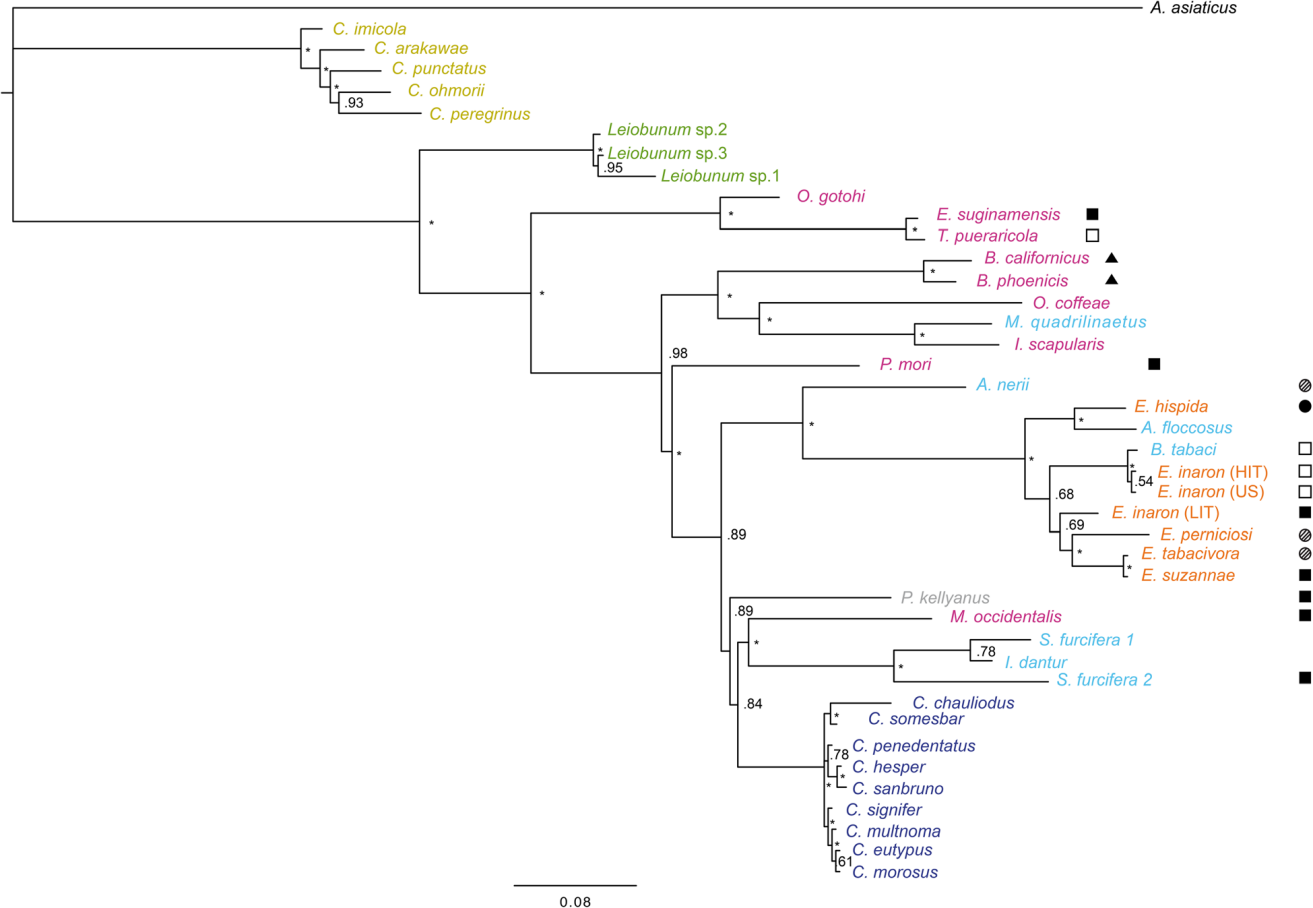
Bayesian phylogeny with of all *Cardinium* strains from this study using concatenated loci: *gyrB*, *sufB*, *EF-G*, and *groEL*. Node support of > 0.99 posterior probability is indicated by an asterisk. *Cardinium* strains are labeled by the host taxon species name and colored by the host taxon order or sub-class. Acari are pink, Diptera are mustard yellow, Opiliones are green, Thysanoptera are grey, Hemiptera are light blue, Hymenoptera are orange, and Araneae are deep blue. Symbols refer to reproductive phenotype when it has been investigated: filled squares indicate cytoplasmic incompatibility (CI) has been shown, empty squares indicate CI has been looked for and not found, filled triangles indicate feminization, filled circles indicate parthenogenesis-induction has been shown, and hatched circles indicate an association with a parthenogenetic host

**Fig. 2:**
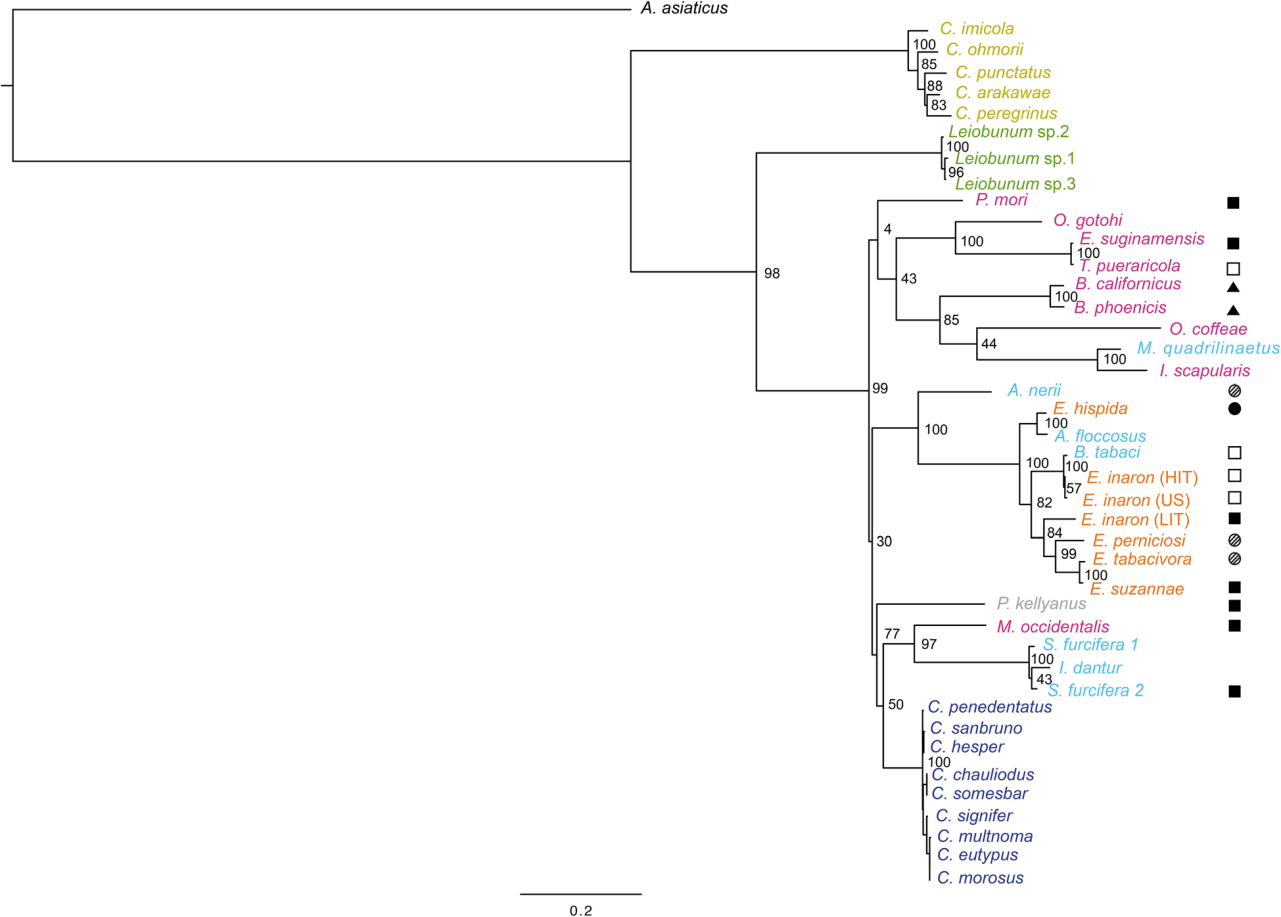
Maximum likelihood phylogeny with of all *Cardinium* strains from this study using concatenated loci: *gyrB*, *sufB*, *EF-G*, and *groEL*. *Cardinium* strains are labeled by the host taxon species name and colored by the host taxon order or sub-class. Acari are pink, Diptera are mustard yellow, Opiliones are green, Thysanoptera are grey, Hemiptera are light blue, Hymenoptera are orange, and Araneae are deep blue. Symbols refer to reproductive phenotype when it has been investigated: filled squares indicate cytoplasmic incompatibility (CI) has been shown, empty squares indicate CI has been looked for and not found, filled triangles indicate feminization, filled circles indicate parthenogenesis-induction has been shown, and hatched circles indicate an association with a parthenogenetic host

**Fig. 3:**
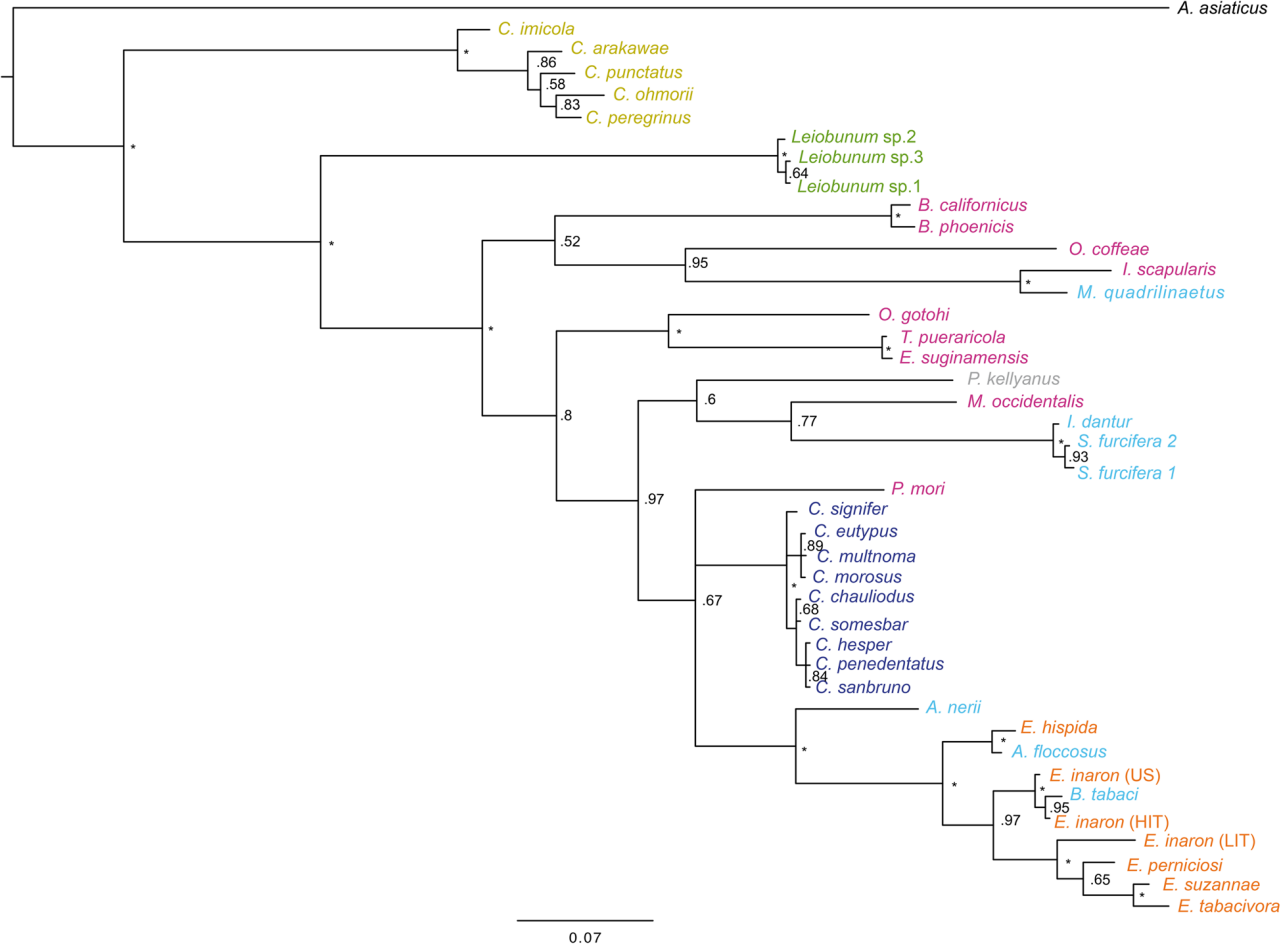
Bayesian single gene tree of 482 bp of Translation Elongation Factor G (*EF-G*) of all strains in this study. Asterisks indicate > 0.99 posterior probability. *Cardinium* strains are labeled by the host taxon species name and colored by the host taxon order or sub-class. Acari are pink, Diptera are mustard yellow, Opiliones are green, Thysanoptera are grey, Hemiptera are light blue, Hymenoptera are orange, and Araneae are deep blue

**Fig. 4:**
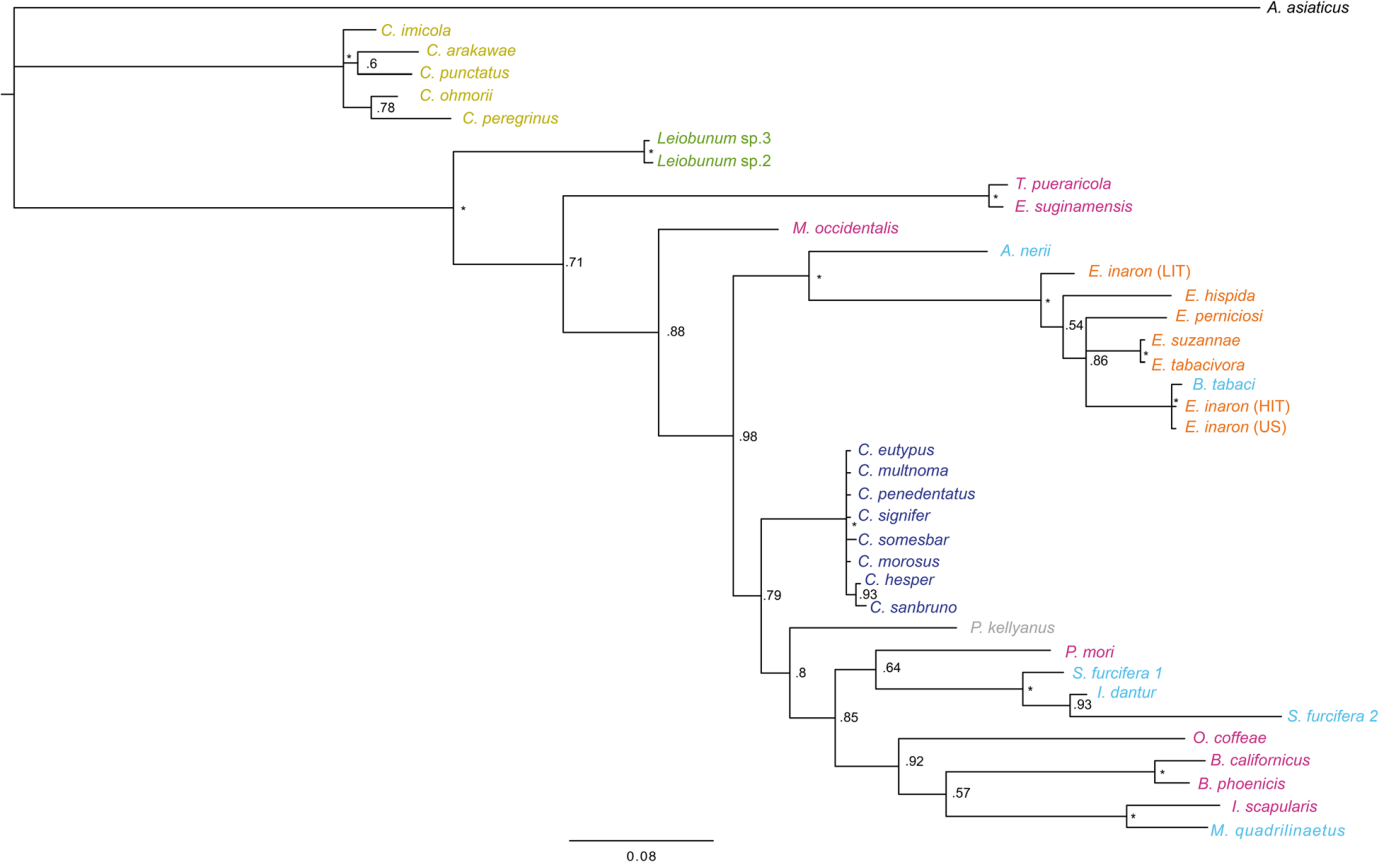
Bayesian single gene tree of 452 bp of Iron Sulfur Cluster Assembly Protein B (*sufB*) of all strains in this study. Asterisks indicate > 0.99 posterior probability. *Cardinium* strains are labeled by the host taxon species name and colored by the host taxon order or sub-class. Acari are pink, Diptera are mustard yellow, Opiliones are green, Thysanoptera are grey, Hemiptera are light blue, Hymenoptera are orange, and Araneae are deep blue

**Fig. 5:**
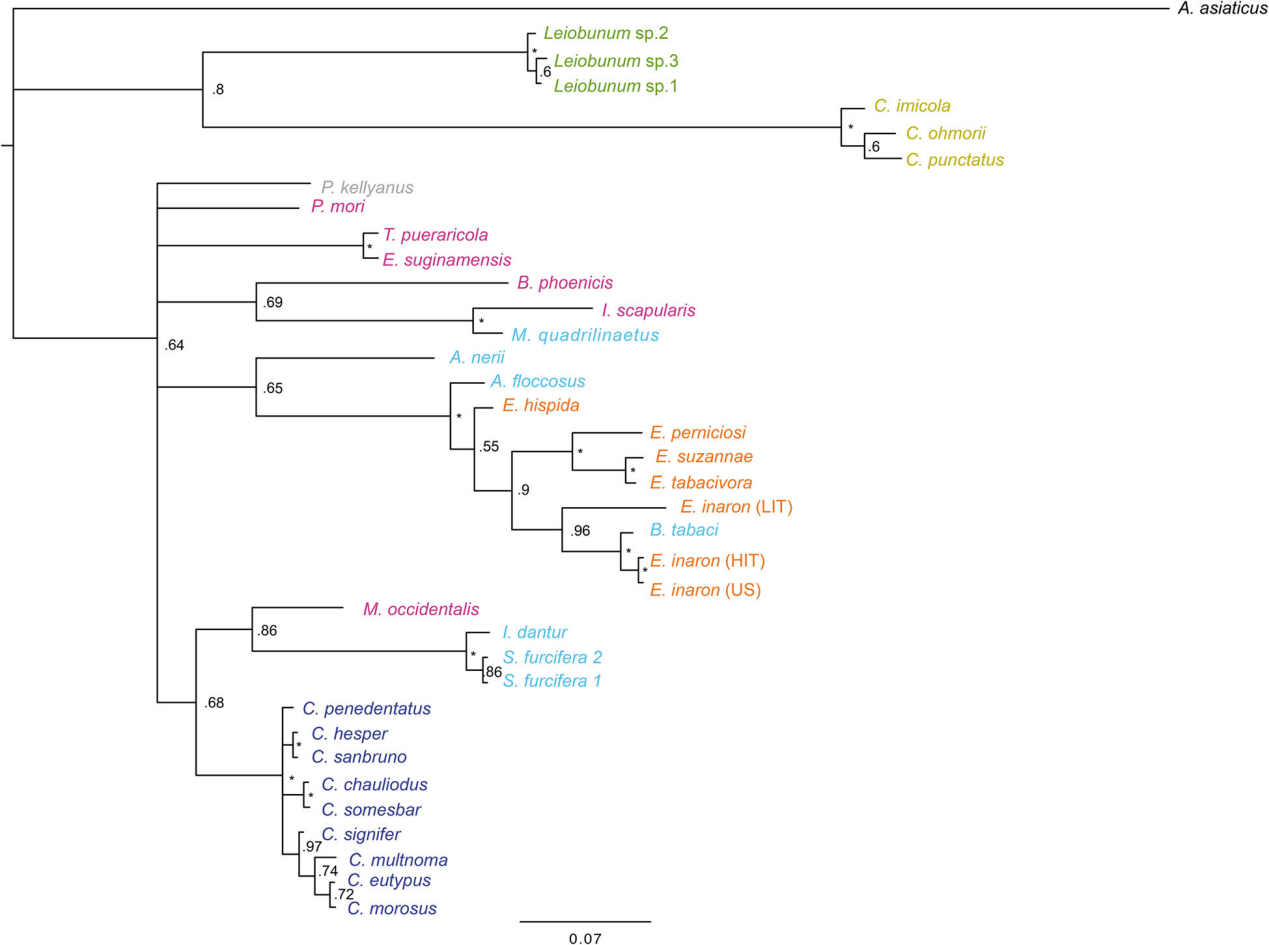
Bayesian single gene tree of 476 bp of gene coding for heat shock protein GroEL of all strains in this study. Asterisks indicate > 0.99 posterior probability. *Cardinium* strains are labeled by the host taxon species name and colored by the host taxon order or sub-class. Acari are pink, Diptera are mustard yellow, Opiliones are green, Thysanoptera are grey, Hemiptera are light blue, Hymenoptera are orange, and Araneae are deep blue

**Fig. 6:**
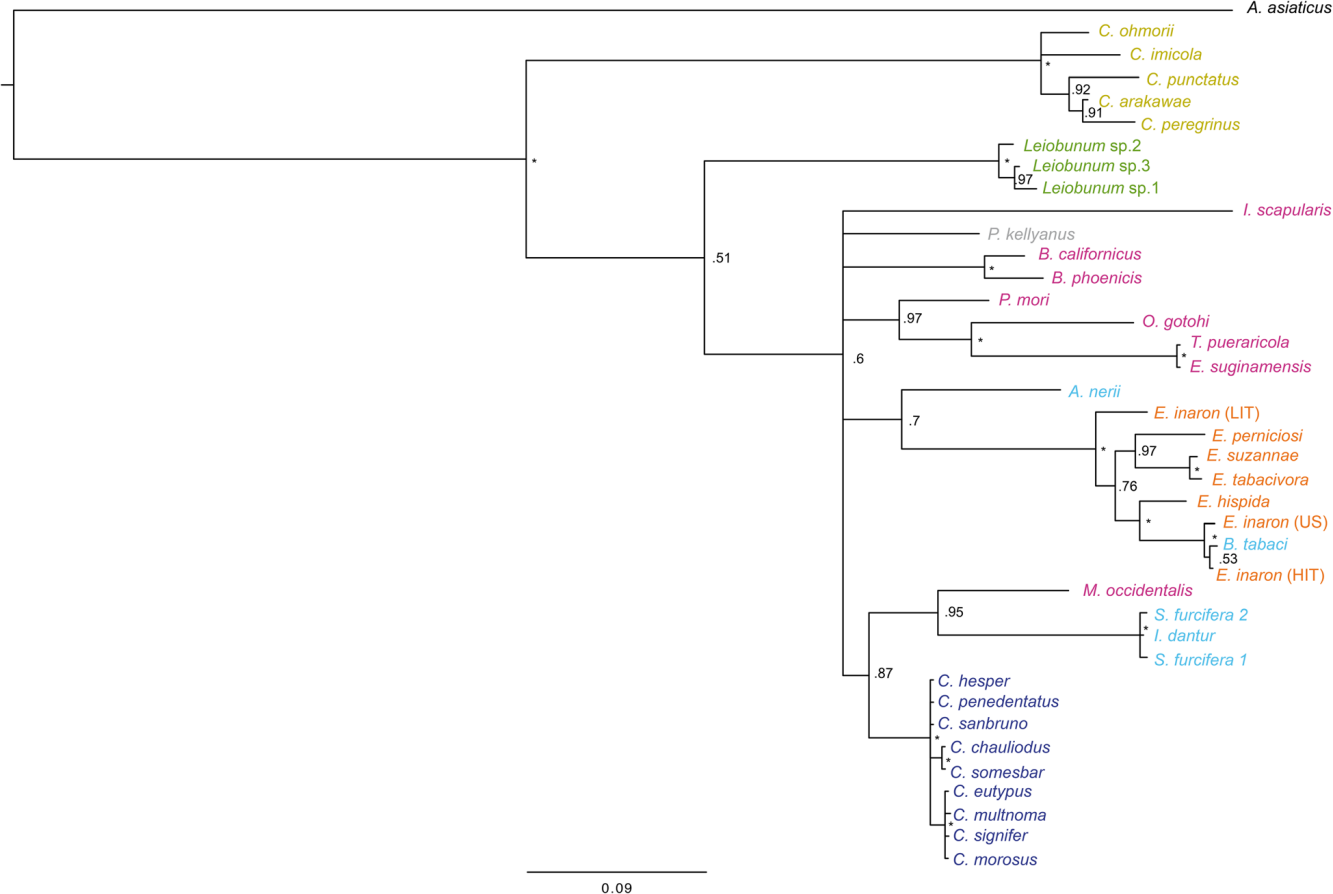
Bayesian single gene tree of 736 bp of Gyrase B (*gyrB*) of all strains in this study. Asterisks indicate > 0.99 posterior probability. *Cardinium* strains are labeled by the host taxon species name and colored by the host taxon order or sub-class. Acari are pink, Diptera are mustard yellow, Opiliones are green, Thysanoptera are grey, Hemiptera are light blue, Hymenoptera are orange, and Araneae are deep blue

The phylogeny of concatenated MLST loci supports the monophyly of *Cardinium* as a genus (Figs. 1 and 2). While the individual gene trees are not completely topologically congruent (Figs. 3, 4, 5, and 6), all phylogenies suggest that groups A and C are each supported as monophyletic groups, as proposed by Nakamura et al. [24]. In addition, the suggestion that group E, with hosts in the Opiliones, is a separate clade [23] is also supported by both individual gene trees as well as the concatenated tree. Evidence of host affinity of related *Cardinium* strains is also shown across phylogenies. This is shown particularly in group A in the *Cebaeus* spider clade, and in a smaller clade showing the sister relationship between strains in the two mites, *E. suginamensis* and *T. pueraricola*. Further, group C is now populated entirely by *Culicoides* hosts, and group E contains entirely Opiliones hosts.

## Discussion

This study aimed to better understand the evolution of the diverse arthropod symbiont *Cardinium*, and provide genetic tools to better identify individual strains within this group. Phylogenies based on sequences derived from four loci across a representative set of *Cardinium* strains show a greater resolution of *Cardinium* clades in this diverse genus than single gene trees using more slowly evolving DNA such as the 16S rRNA gene.

Direct sequencing and analysis of the genes selected for the MLST and phylogenetic analyses suggested that they were almost always single copy genes, although there appeared to be two copies of *SufB* in one host species, the planthopper *Sogatella furcifera.* The single gene trees were not entirely congruent with each other (Figs. 3, 4, 5, and 6), as is most common in bacterial multi-locus sequence analyses [45], and underscores the value of combining data from multiple genes. There are several potential reasons for non-congruence of gene trees and lineage trees [46]. They include lateral gene transfer, which is common in bacterial endosymbionts. However, none of the MLST genes are among the 68 *Cardinium* genes that showed evidence of horizontal transfer in the *Cardinium* genome *c*Eper1 [37]. Lineage sorting, where polymorphisms in a gene precede the lineage split is another possible cause of non-congruence, although lineage sorting is more likely when population sizes are large [46], and endosymbionts typically have small effective population sizes [2]. Lastly, gene duplication and extinction is another process that can lead to non-congruence of gene trees with lineage trees, since different loci may be represented in different terminal taxa, even in single copy genes [46].

Using 16S rDNA and gyrase B, Nakamura et al. [24] grouped *Cardinium* into three groups: A, which contains *Cardinium* strains infecting insects, mites, and other arthropods, B, which contains the *Cardinium* strain infecting the plant parasitic nematode, *Heterodera glycines*; and C, which contains *Cardinium* infecting biting midges in the genus *Culicoides*. These groups are supported in the current study using the concatenated sequence of four loci. Chang et al. [23] suggested that the *Cardinium* found in in the harvestmen clade (*Leiobunum* spp., Opiliones) might be an independent group, based on a phylogeny constructed using a partial 16S rRNA sequence. However, because the 16S rRNA gene displays a relatively slow rate of evolution, the phylogeny provided limited support for this idea. The current phylogeny using the concatenated loci provides more robust support for a separate clade of *Cardinium* found in the *Leiobunum* Opiliones. Following the convention of Nakamura et al. [24] this clade is designated group E, with clade D reserved for *Cardinium* in *Daphnia,* water fleas [47].

The monophyly of *Cardinium* in the oleander scale, *Aspidiotus nerii* (Diaspididae), *Encarsia* parasitic wasps, and whiteflies is supported. Species of *Encarsia* that harbor these *Cardinium* parasitize either whiteflies (*E. hispida*, *E. suzannae*, *E*. *tabacivora*, *E*. *inaron* (IT and US)) or armored scale insects in the Diaspididae (*E*. *perniciosi*). The placement of these *Encarsia Cardinium* strains with those from scale insects (*A. nerii*) and whiteflies (*A. floccosus*, *B. tabaci*) suggests that horizontal transmission events between host and parasitoids, and perhaps among parasitoids, have occurred, although the directionality of these events can only be discerned with a deeper sampling of both hosts and parasitoids. Additionally, none of the closely related strains of *Cardinium* residing in whiteflies, and two related species of *Encarsia* (*c*BtQ1, *c*Eina1, *c*Eina2) are known not to cause CI or any other reproductive manipulations [48, 49]. This suggests that the ancestral strain of *Cardinium* in this group either spread with a phenotype other than some type of reproductive manipulation, or lost its ability to manipulate in each new host after it spread. The *Cardinium* group in this clade is the clearest example of closely related *Cardinium* strains residing in distantly related hosts, in contrast to the previously observed pattern of closely related *Cardinium* strains residing in closely related hosts [24, 50–52], a pattern which is generally supported in the phylogenies of the current study as well.

Interestingly, some patterns appear at least superficially similar between *Cardinium* and *Wolbachia*. The reproductive manipulations that *Cardinium* is able to induce overlap with *Wolbachia*. Strains that cause the same reproductive manipulations do not clearly form one monophyletic clade, except perhaps in the case of the mite strains causing feminization, but this might change when further examples of feminizing *Cardinium* are discovered. Additionally, closely related *Cardinium* strains do not necessarily cause the same reproductive manipulations, as exemplified by the sister strains *c*Eper1, which causes CI, and the parthenogenesis-inducing (PI) strain, *c*Eper2 [25, 30]. Similarly, *c*Esug1, which causes CI, and *c*Tpue1, which does not cause CI or PI, are sister taxa [29]. This pattern also occurs in *Wolbachia*; closely related *Wolbachia* strains in *Acraea* butterflies have shown multiple transitions between sex ratio distorting and CI-inducing *Wolbachia* strains [53]. Additionally, in *Drosophila*, *w*Mel, causing CI, and *w*Au, having no phenotype, are also very closely related [54]. These similar patterns between *Wolbachia* and *Cardinium* trees are not necessarily expected; recently, it has been suggested that the horizontal transfer of the CI phenotype may be linked to the *Wolbachia*’s WO phage, which can cross-infect *Wolbachia* strains [55, 56]. So far, sequenced genomes of *Cardinium* do not show the presence of phage DNA. Unlike *Wolbachia,* however, many *Cardinium* strains do harbor plasmids [37, 40], which may serve a similar function in horizontal transmission of reproductive manipulation genes [57, 58].

We fully expect the phylogeny of *Cardinium* to become better resolved when more *Cardinium* genomes are published, as have *Wolbachia* genomes [59]. There are currently six *Cardinium* genomes published [37, 40–44], three of them published in the last couple of years, so it is reasonable to expect more in the near future. Indeed, the high genetic diversity within this genus made the design of a single MLST, a scheme designed for strains within a bacterial species [60], challenging. However, while full genomes are always going to be better for inferring phylogenies and group placements [61], the cost of sequencing, the sequencing depth necessary for symbionts that may exist at relatively low titer in their hosts, and the expertise in assembling symbiont genomes from metagenome data can still be a limiting factor for many laboratories. For ecological studies and surveys in particular, the ability to relatively quickly type *Cardinium* strains meets the objective of giving the strain an identity and fitting it into the *Cardinium* phylogeny. This MLST offers a relatively low-cost way to differentiate between strains of *Cardinium* and is a starting point for researchers considering the study of *Cardinium*. In particular, the utility of the *Cardinium* MLST will be valuable in answering questions concerning relatively recent biogeographic or host switching events.

Characterizing a strain of a symbiont with an MLST allelic profile may be difficult when more than one strain co-infects individual host individuals. If both alleles are amplified, direct sequencing may not be possible, and cloning may be required prior to sequencing. Even more challenging is determining which sequenced allele at a particular locus belongs to which strain. There are a couple of potential solutions to this problem. When multiple strains are present in different combinations among individuals, one can logically examine the sets of alleles in multiply infected and singly infected individuals to allow assignment of allelic profiles to strains, a system known as Allelic Intersection Analysis [62]. This may be particularly relevant in complex situations like that found in the apple maggot, *Rhagoletis pomonella,* where up to four *Wolbachia* strains have been found in multiple combinations [63]. Another tool that could be useful when co-infecting symbiont strains are found at different titers is quantitative PCR. It may be possible to design specific qPCR primers for each allele and quantify the relative titer of each. If the titer is consistently higher in one set of alleles than the other, one can presume the alleles in that set belong to the same strain. In the host *Encarsia inaron* (from Italy) coinfecting strains *c*Eina2 and *c*Eina3 are found at high and low titers, respectively (Table 1).

## Conclusion

*Cardinium* evolution appears to be driven by both ecological opportunity and host specialization. *Cardinium* has frequently switched between parasitoids and their hosts, even though they are physiologically quite different, causing these strains to form a clade. In contrast, the *Cardinium* in *Cybaeus* spiders, *Culicoides* spp., and *Leobinium* spp. appear to be quite specialized to particular host lineages, without distantly related hosts breaking up these clades. Similar to *Wolbachia*, the relatedness of *Cardinium* strains does not necessarily predict their reproductive phenotypes. Overall, the new genetic tools proposed in this study allow for clearer strain delimitation and a more detailed picture of the evolution of *Cardinium*, one that will keep unfolding the more the MLST primers are used to characterize strains and add taxa to the *Cardinium* phylogeny.

## Methods

### Gene selection

Four genes with the highest amino acid identity between the sister group to *Cardinium, Amoebophilus asiaticus*, and the sequenced *Cardinium* strain, *c*Eper1, were chosen to develop a Multi Locus Sequence Typing (MLST) system with other strains [37]. We did not attempt to choose genes that are evenly spaced around the *Cardinium* chromosome. While, in more conserved lineages, linkage among loci is often avoided by choosing MLST genes that are evenly spaced [60], in *Cardinium* there is little shared synteny, even between the two related sequenced genomes, *c*BtQ1 and *c*Eper1 [40]. In addition to making even spacing of chosen genes unworkable across the genus, the low level of synteny suggests frequent gene rearrangements in this lineage, and a low probability of linkage among loci. The genes selected for this study were: Elongation Factor G, a protein responsible for coordinating the movement of tRNA and mRNA during translation [64]; gyrase B, a topoisomerase that unwinds DNA during DNA replication [65]; Iron Sulfur Cluster Assembly Protein (SufB), a protein involved in generating Fe-S complexes mainly involved in electron transfer [66] and the Heat shock protein GroEL, a chaperone protein essential in stress-related responses [67].

### DNA extractions

Arthropods with confirmed *Cardinium* infections and DNA samples were received from cooperators around the world (Table 1). From Japan (H. Noda), we received planthopper, mite, and biting midge DNA, extracted as described in Nakamura et al. [24]. *Cardinium* from the *Ixodes* cell line ISE6 (T. Kurrti) was processed by shearing the cells and filtering them through a 1.5 μm syringe, then extracting the lysate with 3 μl of 20 mg/ml proteinase K and 50 μl of water with 10% w/v chelex beads [49]. *Cybeus* spiders (S. Perlman) were extracted using Qiagen DNeasy extraction kits. All other samples of alcohol-preserved specimen were also extracted using the chelex extraction protocol.

**Table 2:** MLST primers and their suggested melting temperatures for PCR

Primer name	Primer sequence (5′ – 3′)	Tm (°C)	Gene length (bp)	Amplified nucleotide range of gene (bp)	MLST fragment size (bp)
gyrb_859F	ATGCAYGTMACBGGDTTTARAAG	50	1950	859–1637	736
gyrb_1637R	TARAGTGGRGGRGARGCAAT				
groel_346F	VTHAARCGBGGBATWGACAA	52	1638	346–842	476
groel_287F^a^	CNCARKCTATWTTYRYVCATGG				
groel_842R	TTGGBGAYAGAAGRAARGCNATG				
sufb_806F	CTACNGTDCARAATTGGTATCC	50	1443	806–1289	451
sufb_1289R	ADYTGRTCYKCRCTRATTTT				
EF_1689R	AAABCCYTTYTGAATIGCTGG	52	2142	1689–1162	482
EF_1162F	GCNGTRGTIGGITTTAARGARATTA				

^a^Alternative forward primer for *groEl*

### Primer design, PCR, and sequencing

Primers were iteratively designed as sequenced products from strains were added to sequence alignments. Initially, general primers were designed based on the only two sequenced (and closely related) *Cardinium* strains (*c*Eper1, *c*BtQ1) and the sister taxon to *Cardinium*, *Amoebophilus asiaticus 5a2*. These initial primers were designed using *c*Eper1 as the reference strand in Primer3 [68, 69] with ambiguities based on the other strains added manually. Amplification of some gene products was not successful from all strains using these initial primers, particularly from strains divergent with respect to *c*Eper1 and *c*BtQ1, such as those in the biting midges, *Culicoides* spp. In these instances, strain-specific primers were designed once a small segment of the gene was sequenced. These strain-specific primers were then used in conjunction with the initial degenerate primers to obtain more sequence. When more than three bacterial strains were used for primer design, areas of conservation were manually detected and these potential primer regions were checked for hairpins and tendency to form primer dimers in Primer3 [69] against every strain. All primers were selected by minimizing the number of ambiguities and maximizing the number of conserved base pairs in the 3′ primer region, and M13 tags were added to the primers for ease of sequencing [70].

Although the melting temperature varied depending on the primer pair (Table 1), PCR conditions were generally as follows: 15 μl reaction volume with New England Biolabs buffer and Taq at 1X concentration, 5 mM dNTPs, 0.76 mM MgCl_2_, 1.1 μM primers with 2 μl of DNA. From mite extractions, 4 μl of DNA was added (similar to Groot and Breeuwer (2006)). The initial melting temperature was 94 °C for 2 min; this was followed by 40 cycles of 94 °C for 45 s, the annealing temperature (Table 1) for 45 s, and extension at 68 °C for 45 s. The final extension was at 68 °C for 7 min.

### Phylogenetic analysis

DNA sequences were quality-controlled and aligned using CLC Main Workbench 6 (Qiagen) and MUSCLE [71]. jModelTest was used to select the optimum model of evolution based on the Akaike information criterion [72]. Bayesian trees were constructed in MrBayes with one million Markov Chain Monte Carlo (MCMC) generations and sampled every 1000 generations [73]. Maximum likelihood trees were constructed using RaxML with 1000 rapid bootstraps. Both Bayesian and ML methods used the GTR + I + G model of nucleotide evolution with a total of 2145 bp from Gyrase B (*gyrB*), translation elongation factor G (*EF-G*), Iron Sulfur cluster assembly protein (*sufB*), and heat shock protein (*groEL*) for each taxon, partitioned by gene and codon position. Phylogenetic tree figures were generated in Mesquite [74].

## Data Availability

The sequence datasets generated and/or analyzed during the current study have been deposited in the NCBI repository, under accession numbers MK264778-MK264911 [https://www.ncbi.nlm.nih.gov/nuccore/].

## Abbreviations

CI: Cytoplasmic incompatibility
MCMC: Markov chain Monte Carlo
MLST: Multi-locus sequence typing
PCR: Polymerase chain reaction
PI: Parthenogenesis-induction
qPCR: Quantitative polymerase chain reaction
